# What a Fool!

**Published:** 1868-02

**Authors:** 


// HALL'S JOURNAL OF HEALTH.

Our Legitimate Scope is almost boundless; for whatever begets pleasurable and harmless feelings, promotes Health; and whatever induces disagreeable sensations, engenders Disease.

WE AIM TO SHOW HOW DISEASE MAY BE AVOIDED, AND THAT IT IS BEST, WHEN SICKNESS COMES, TO TAKE NO MEDICINE WITHOUT CONSULTING A PHYSICIAN.

VOL. XV.] FEBRUARY, 1868. [No. 2.

### "WHAT A FOOL!"

Is an inquiry which many a reader has made of himself, quite a number of times in the course of his life; not for the purpose of information, but as a means of expressing his strongest conviction that he himself was the most insensible simpleton within his knowledge; and it is a fact, that the wisest of men do commit acts which are perfectly unaccountable except on the supposition that there are moments in the life time of all, when the mind is bereft of its reasoning powers; when its rationality is in abeyance; whoever has had no such experience let him turn to some other article, with the congratulation of having companionship with that most graceless scamp of Scripture record who asserted it as his conviction that he "was not as other men are." As for ourself, we own up, in manner and form following, to wit:

At daylight on a December morning, we found ourselves in night-gown, pants and India-rubbers, standing in the kitchen door surveying our plantation of one thousand——square feet, covered with half a yard of snow, and the following thoughts ran through the brain in less than half a minute; this is washing day, and will be one of cloudless sunshine; but the flags are covered with snow and our girls must either remove it, or trample through it all day, in either case, involving cold, wet shoes and wet feet, threatening severe colds, sickness and perhaps protracted suffering, if not death itself; now if we have any hobby on the face of the earth, it is the avoidance of sickness, whether as to ourself or others, because it is such a trouble to be sick; it deranges the whole household, and imposes additional and unpleasant labor on every member of the family; to say nothing of that wearing solicitude which eats out every domestic enjoyment and engenders an atmosphere of sadness and gloom; of uncertainty and foreboding, which waste the strength, wear out the body and press upon the spirits with an intolerable weight; in the case in hand, the shortest, safest and best course would be for us to remove the snow ourself; and why not? we were young and vigorous; it would not take long, and it would be such a help to the girls; the strength which they would expend on that unusual work, if employed in rubbing the linen, would be an advantage to our three young daughters and our youthful son and heir; and here peeped in that miserable little elf self; "if our girls get sick we might waste months in finding two more, any thing like as good," for two good servants in any one family in New York is the highest prize in any lottery, and we have more; they need no scoldings from one month's end to another, and there is not a loud, angry word heard in our household; now is it not worth while to be considerate, and keep our "help" under such circumstances? And so, although the thermometer was about twenty above zero, and a strong cold west wind was blowing, we went to work, and when it was completed, the whole body was in a profuse perspiration; but the neck, throat, and upper part of the chest felt as if they were frozen a foot, more or less, deep; and then came out the expression, with very considerable unction, "What an unmitigated fool have I been, to be working all this time with head, and neck, and chest exposed to such a piercing cold wind!" Then came up visions of croup, diptheria, quinzy, putrid sore throat, and a dozen other hob-goblins, any one of which might have put us past cure within forty-eight hours; and already there was inability to draw a full, deep, satisfactory breath; and now, reader, for the idea which we wanted to impress on your mind, at the expense of being thought a very egoistic individual; but if we can put you in a way of saving your in—? valuable life! we will feel well compensated. The only course to pursue was to keep up a vigorous circulation, so as to prevent chilliness; for if that had taken place, pneumonia would have been a pretty certain event; but exercise for this purpose was out of the question, because we were already jaded out; perhaps a non-te-totaller would have resorted to a deep and hearty swig of wiskey; but that was not in our line, and besides there was not a drop in the house, then there remained but one means left, the application of heat, by means of fire and hot water; so we paddled the hands and face in water as hot as could be borne, and it was exceedingly grateful; then dressed as rapidly as possible and sat by a red-hot kitchen range, until the whole body was perspiring again; took breakfast, and went to writing an article for the Journal about murdering children in the public schools. As far as we know, we did not suffer the slightest injury by the unwise exposures which have just been detailed. Perhaps the first thought of the reader would have been to have applied the hot water to the neck, throat and upper part of the chest; that would have been inconvenient, would have been but partial, and would have dribbled the upper end of the flannel shirt, which was already wet with perspiration and was also very cold, but the hands and the face were at the extremities, and hot water would bring the blood to them, through the parts which seemed to be so cold, and thus warm them up in its progress; the hands could be kept continously hot, by keeping them immersed in hot water; the same with the face; but to apply water to the neck, with the hand or sponge, would be but for an instant, and only for the space which was covered by the hand, and it may be useful to remark here, that the safest way to cool off, even in summer, is to dip the hands in hot water, and raise them in the air; do this successively; the philosophy of it is, that the evaporation of heat is very rapid, and is carried off by the steam caused by the hot water and warm skin; cold water will do the same thing, because the heat of the skin converts it into steam, but it is a harsh method, and is never so safe; and many times, when a person comes into the house from a cold walk or ride, feeling as if a chill were iminent, a most comfortable method of averting the chill, and of warming up the whole blood, is to immerse the hands in hot water, and keep them in motion; for if allowed to remain still, the layer of water next to them becomes in a measure cold; while by moving them about, the skin finds new layers of hot water, and thus every motion is grateful· The same may be said as to the feet; it is certainly a very agreeably method of getting warmed up; and when you have become so, it is well to dip both hands and feet into a vessel of cold water, for a second or two. Of course, if the extremities are frozen, stiff with cold, or without feeling, then snow or very cold water should be applied under the supervision of a physician.

But was it not undignified for us to be shovelling snow in trowserloons and shoes? we do not consider any thing "infra—dig" which promotes health and is a help to others. Trousseau, one of the greatest medical men of his age, whom kings and queens, within ten years, have been glad to have it in their power to consult, tells us that he brought on a very severe attack of asthma by concealing himself in the hayloft to see whether his coachman did not steal his horse feed.

The great Newton was found once in a less dignified employment, smoking a vulgar pipe, puffing away for life, like a young steam engine drawing smoke in and then as soon as it got it in, go about hustling it out and this for a whole hour at a time; and what's the use of it? it makes you no wiser and no better; you infume your whole clothing, you spit and hawk and skatter around your saliva, on carpet, floor furniture, and the dress of your friends; bespattering your own clothing with nauseous looking stains, and often having a streak of filthy slaver extending from the corners of the mouth, and than to find ourself such a helpless slave to the beastly custom; but this is off the subject; Sir Isaac's sweetheart is reported to have been sitting by him, and wishing to adjust the fire of his pipe he took her finger, instead of his own, and so burned it, as to outrage her feelings, and she never would have any thing to do with him more; so we think our action in the premises will favorably compare with that of the great Physician or the greater Philosopher. But let us here tell a valuable secret as to the manner in which great men employ their time.

No man can study advantageously all the time; there must be some relaxation, or the brain will become disorganized, or otherwise hopelessly diseased. No man ought to study hard on one subject more than four hours a day, and give ten hours to purposes of dressing, sleeping and eating, leaving ten hours for mental recreation, that is, mental rest, which is done in two ways: first, engaging the mind in thinking about something else; that is, putting other organs of the brain to work; second, engaging in muscular motion, which does good in two ways; it works out of the system the waste particles made by hard thought, and thus purifies the blood, fitting it for building up the brain, repairing its wastes, making it ready for new work. But muscular motion does good in another way; the mind is diverted to it, and is thus rested from the main study; hence the true policy of hard students is not to sit, or loll, or lounge about, but to be doing something with the hands or feet which is of sufficient interest to engage the mental notice, and, if pleasurably engaged, so much the better, even by fifty or a hundred fold, hence the three employments, or side works, of the three great men named were really mental rests, recreations, whether it was Trousseau playing watchman in his hay-loft, or Newton feeling his pipe with his sweetheart's finger, or the editor of Hall's Journal of Health! clearing the flags of snow in shirt and pants at daylight, of a December morning, for an hour or two, for the benefit of two good, tidy-looking, young, unsophisticated servant girls, who had never "worked out" before. We rather think that our mode of resting the mind was the most utilitarian, plilosophical, humane, and least wickedly risky of the three.

It will be useful to remark here that we do not like to use our eyes in reading or writing until breakfast has been eaten, because they are stronger all the day afterwards; nor do we use them in that way after twilight; this has been our uniform habit, with the result, as we think, that we were able to do without the use of glasses for eight or ten years after our old school-mates of the same age had been using them, and we are writing now without any glasses whatever. Albert Barnes, the eminent commentator, rose habitually at four o'clock for study, and soon had to abandon all study, go abroad, and, after years of lost time, is prematurely laid on the shelf from diseased eyes.

Literary and professional men, in consulting us, often inquire, with great earnestness, how can we take exercise in a large city, or town or village—"there is nothing that we can do." In the first place, eat about one-half less every day, and you will at once require but half the amount of exercise; now for the remainder. Have you a family? If you have not, you need not take any exercise, and you can eat, and stuff, and guzzle all day, for you are of no account, and the sooner you die off and make room for a better man, the better for society at large. But taking it for granted that you have a family, like other respectable men, and live in a brown stone house on Murray Hill, New York, there are a multitude of very useful things you can do every day to the comfort of your family, the benefit of your health, the improvement of your digestion, the soundness of your sleep, the vigor of your thought, and the benignity of your disposition, get up at five o'clock winter and summer, go down into the cellar, riddle out all the cinders of the day before from range, furnace and grates, sprinkle them with water, and put them in the furnace; if you are hardy and systematic you can do all this in half an hour, and save about half a dollar besides, if you have a good-sized family; next help your servant girls, giving them a chance to sleep a little longer, by kindling a few of the fires, and, if you are handy, you will save a good deal of paper and wood-kindling every day.

Literary and professional men maintain an idle theory when they consider every moment lost which is not employed in reading, writing, or investigation; it is loss of time in the long run which should alarm the individual; it is the curtailment of human life for ten, twenty, and even thirty years, which should startle the mind and lead to a wiser way of life. Whoever indulges in brain-work over four hours a day habitually, does in proportion shorten his life, or at least shortens the term of his usefulness; this is a great general rule, to which there may be some exceptions; but let the reader take it for granted that he is not one of those exceptions; on the contrary, whatever of time spent in muscular activities, beyond the four hours of brain work, adds that much to the probabilities of a longer life, and a life, too, of greater efficiency.

### Brain and Body Work.

Physiologists, after patient and close inquiry, have arrived at the important and practical conclusion that the power of the entire man, his vitality, is as much expended by two hours of deep mental effort as by a whole day of ordinary bodily labor; this fact seems to be founded on observed physiological laws; hence, the man who spends four hours in the twenty-four in earnest mental labor, goes to the utmost allowable limit for a day's work, and all the time that remains, after deducting ten hours for eating, sleeping and dressing, should be conscientiously expended in muscular exercises which require no special brain effort, and such exercises should always, by preference, be those which are agreeable, useful and profitable; for they not only promote the healthful condition of the body, but give rest to the brain, which, by that rest, recuperates its powers ; many can remember, when turning back to their school-days, that they have gone to bed feeling that they did not know their lessons, yet, on rising in the morning, the mind would run over them with a gratifying and surprising clearness. It is this which accounts for the observation that persons have striven hard to remember some important fact, or as to where valuable papers have been laid, and towards morning, when the mind began to awake, a little before the body, this being the time of dreams, the point is made clear in the form of a dream, thus showing that rest of the brain, whether by actual sleep or the passive, comparative rest which manual labor affords, gives mental activity, vigor, perspicacity; from these it follows that no form of muscular exercise is ignoble in a student, a brain-worker, which has to be done by some one, and by being done by him, will save money, or will save the time of another, who, perhaps, may already be over-taxed. How many servants are over-taxed! how many faithful, uncomplaining wives are over-taxed! and sons and daughters sometimes ; and clerks, and apprentices, and other employees. In every dwelling in a large city, there are many things which the master could do which would reflect benefit on himself and others also; some of these may be suggested : get up by daylight, clear the snow from the sidewalks, kindle two or three fires, ventilate your parlors, keep the cellar well swept, split up kindling-wood, after sawing it yourself ; whitewash the cellar twice a year, as also the fencing around the back-yard ; trim the eight or ten grape vines which you ought to have against the fence ; kill off the worms which infest them in the summer ; root out the clover and weeds from your grass plat ; keep your hundred feet of flower-borders in perfect order ; if you have a library, dust your books, rearrange them so that you may be able to put your hand upon them in the dark if needed ; assort your pamphlets and magazines, so that no time may be lost should you want any of them in a hurry ; in this way valuable time may be saved on occasions when you have no time to spare ; then pump water in your tank for twenty minutes every morning.

tank for twenty minutes every morning ; repair all the broken glass yourself ; learn how to keep all cracks in the plastering filled in with plaster of Paris ; keep your roof well painted ; and you great big lazy hulk you, why might'nt you as well, when all these things are done, and you have any unoccupied time, help your wife darn some of the basket full of stockings ; or sew on the lost buttons of your boys who are at school ; or trim their hair, and sew up the rips in shoes, and cut down the pegs or tacks in the inner soles, which so often do permanent injury to the feet ; keep their skates in order ; have a grindstone of your own and an iron vice, and keep all the knives sharp, and the handles tight, learn how to mend broken china and common delf ; to tack down carpets ; to hang pictures ; to take stains out of marble and wood ; to replace mahogany veneering ; to render chair legs and backs firm ; to keep the tubs and barrels hooped up ; learn how to make good flour paste, and keep some always on hand, ready to mend a torn bank note ; or paste a useful newspaper scrap in some appropriate place ; or have a book for domestic receipts, and when you see one which seems to be valuable, paste it in the book under its proper alphabetical head. What a marvellous help any husband might be to his wife and family in ways like these, and be saving many a dollar besides, instead of lolling about on the sofa or chairs; or, with feet on table or mantle, leaning back and smoking a filthy pipe or noisome cigar, or sipping the murderous brandy and water, or vulgar " Lager ;" or wasting time in pitiful card playing or childish checkers or chess or back gammon or solitaire, or any other useless time-murdering, or mind dwindling occupation ; or take a good long walk after tea, with yourself, wife and children to some profitable lecture ; to some prayer meeting, or other useful assemblage of the good, keeping diligently away from the theatre, the dance and the club house, all three, the equal destroyers of social purity, of domestic happiness and family elevation. Fathers, mothers, husbands, wives, think of these things, and be encouraged to do them, by the reflection that the editor of Hall's Journal of Health has been practising thus for many years and keeps young and thrives upon the same in physical well-being and is as lively as a cricket and lithe as a lark, while all his college cotemporaries have grown old and gouty and string-halt and stiff, or have laid down to rest in the peaceful grave ; he the only one of all his class who stands in his lot fit for the duty of one man, and doing that of three. But who knows how soon it will be all over ? Next year, next week, to morrow! for "we are all as a vapor that appeareth for a little while, then vanisheth away," such shadows we are, such shadows we pursue

